# Regional cerebral THK5351 accumulations correlate with neuropsychological test scores in Alzheimer continuum

**DOI:** 10.22038/AOJNMB.2022.67827.1470

**Published:** 2023

**Authors:** SungWoon Im, Kohei Hanaoka, Takahiro Yamada, Kazunari Ishii

**Affiliations:** 1 Department of Radiology, Kindai University Faculty of Medicine, Osakasayama, Osaka, Japan; 2 Institute of Advanced Clinical Medicine, Kindai University Faculty of Medicine, Osakasayama, Osaka, Japan

**Keywords:** Alzheimer's disease, Fluorodeoxyglucose, Amyloid, Tau, THK5351, PET, MMSE, ADAS

## Abstract

**Objective(s):**

We evaluated the relationship between regional accumulations of the tau positron emission tomography (PET) tracer THK5351 and cognitive dysfunction in the Alzheimer's disease (AD) continuum.

**Methods:**

The cases of 18 patients with AD or mild cognitive impairment (MCI) due to AD who underwent three-dimensional MRI, fluoro-2-deoxyglucose (FDG)-(PET), Pittsburgh compound B (PiB)-amyloid PET, and THK5351-tau PET were analyzed. Their mean age was 70.6±11.3, their mean Mini-Mental State Examination (MMSE) score was 22.3±6.8, and their mean Alzheimer Disease Assessment Scale-Cognitive Subtest (ADAS) score was 12.5±7.3. To determine the correlation between each patient's four imaging results and their MMSE and ADAS scores, we performed a voxel-wise statistical analysis with statistical parametric mapping (SPM).

**Results:**

The SPM analysis showed that the bilateral parietotemporal FDG accumulations and MMSE scores were positively correlated, and the bilateral parietotemporal FDG accumulations were negatively correlated with ADAS scores. There were significant correlations between bilateral parietotemporal and left posterior cingulate/precuneus THK5351 accumulations and MMSE/ADAS scores.

**Conclusion:**

In the AD brain, THK5351 correlates with neuropsychological test scores as well as or more additional than FDG due to its affinity for both tau and monoamine oxidase-B (MAO-B), and measurements of THK5351 may thus be useful in estimating the progression of AD.

## Introduction

 The U.S. National Institute on Aging-Alzheimer's Association (NIA-AA) Joint Working Group published diagnostic criteria for Alzheimer's disease (AD) in 2011, and since then there has been a move toward the use of biomarkers (e.g., amyloid   and neuronal injury) to strengthen the diagnosis of AD (1). 

 The NIA-AA has classified AD into three stages: 1) AD dementia, 2) mild cognitive impairment (MCI) due to AD, and 3) the preclinical stages of AD. The presence of tau protein was originally included in the description of neuronal injury, but since 2018, AD has been stratified by "ATN" (A for amyloid, T for tau, and N for neuronal injury) in the continuing efforts to understand the disease progression of AD, and tau deposition is now described as independent from neuronal injury (2, 3).

 Tau positron emission tomography (PET) was developed to measure tau deposition in the brain as a biomarker of AD. Regional cerebral tau deposition increases with the AD prognosis and correlates with the degree of cognitive decline, and it was reported that the regional uptake of flortaucipir, one of the first-generation tau PET tracers, is correlated with cognitive tests (4). Another first-generation tau PET tracer, THK5351, was later found to have an affinity for monoamine oxidase-B (MAO-B) and is considered to have off-target issues. We conducted the present study to determine the relationships between each ATN biomarker and neuropsychological test scores in patients on the AD continuum who each underwent PET examinations that used fluoro-2-deoxyglucose (FDG), amyloid, and THK5351.

## Methods

***Patients***

 The present investigation was a sub-study of our global research regarding the pathophysiology of mild dementia as assessed based on Pittsburgh compound B (PiB) and THK5351 PET examinations, which were approved by the Ethics Committee of Kindai University Faculty of Medicine. Written informed consent to participate and for their images and data to be published was obtained from all of the participants. Eighteen patients on the AD continuum (14 patients with AD and four patients with MCI due to AD) who underwent three-dimensional MRI (3D-MRI), ^18^F-FDG-PET, ^11^C-PiB-PET for amyloid imaging, and ^18^F-THK5351-PET for tau imaging were selected. All 18 patients had undergone the Mini-Mental State Examination (MMSE) and had taken the Alzheimer Disease Assessment Scale-Cognitive Subtest (ADAS). Their clinical diagnoses were made with reference to the criteria of the NIA/AA for AD (5) and MCI due to AD (6). Amyloid positivity was verified with a subsequent PiB amyloid PET examination.

 PET images were obtained using an ECAT Accel or Biograph Duo scanner (Siemens Medical Solutions, Erlangen, Germany) or Discovery PET/CT 710 scanner (GE Healthcare, Milwaukee, WI, USA).

***FDG-PET***

 For the FDG-PET, the patient was asked to lie quietly in a dimly lit room with their eyes open and minimal sensory stimulation after having fasted for ≥4 h. At 30 min after an intravenous injection of 185 MBq of ^18^F-FDG was administered, a 30-min emission scan was acquired. Prior to the injection of FDG, blood glucose measurements were performed in all patients to ensure that blood glucose levels were normal (<150 mg/dL).

***PiB-PET***

^11^C-PiB for amyloid imaging was synthesized at the PET Center of our institution as described (7). For each patient's PiB-PET examination, an intravenous injection of a mean dose of 555 MBq (11.1 MBq/kg body weight) of ^11^C-PiB was administered, and 50 min later a 20-min emission scan was acquired (8).

***THK5351-PET***

^18^F-THK5351 was also synthesized at the PET Center of our institution as described (9). For each patient's THK5351-PET examination, a 20-min emission scan was acquired at 90 min after an intravenous injection of a mean dose of 185MBq of THK5351 was administered (10).

 In all of the above-described PET examinations, a transmission scan was performed for the correction of attenuation before the administration of the tracer. All PET images were reconstructed with an iterative algorithm that provided spatial and axial resolution in the range of 6–8 mm at full-width and half-maximum. Standard uptake value ratio (SUVR) images were obtained by normalizing the tissue radioactivity relative to cerebellar accumulation counts.

***3D-MRI***

 Magnetic resonance imaging (MRI) scanning was performed using a 3T Achieva, 1.5T Intera (Philips, Best, The Netherlands) or 1.5T Signa HDxt system (GE Healthcare). The detailed scan protocol including sagittal T1-weighted 3D whole-brain images are described elsewhere (11).

***Image analysis***

 All of the FDG-, PiB-, and THK5351-PET images were co-registered to the individual patient's 3D-MRI results by using Statistical Parametric Mapping 12 (SPM12: Wellcome Trust Centre for Neuroimaging, University College London, London, UK; http:// www.fil.ion.ucl.ac.uk/spm/software/spm12/). The SPM12 segmentation program was used to segment each MR image into gray matter (GM), white matter (WM), and cerebrospinal fluid. The individual patient's GM image was anatomically normalized to the Montreal Neurological Institute (MNI) space with Diffeomorphic Anatomical Registration Through Exponentiated Lie Algebra (DARTEL). 

 Then, spatial normalization of the FDG-, PiB-, and THK5351-PET images to the MNI space was performed using the individual parameters obtained from the individual DARTEL normalization. All GM, FDG-PET, PiB-PET, and THK5351-PET images were smoothed with an isotropic 8-mm Gaussian kernel in order to increase the signal-to-noise ratio and to compensate for differences in gyral anatomy between individuals. The individual SUVR images of FDG, PiB, and THK5351 were produced by normalizing by the cerebellar gray matter uptake counts.

 We used the SPM12 program to conduct a voxel-wised statistical analysis to determine the correlations between the regional FDG uptake (FDG SUVR), PiB uptake (PiB SUVR), and THK5351 uptake (THK551 SUVR) and the patients' MMSE and ADAS scores. A significant threshold of p<0.001, uncorrected for multiple comparisons, and extent threshold as 300 voxels were applied.

 We also evaluated the correlation between regional FDG SUVR and THK5351 SUVR with regions of interest (ROI), using the EPP-ROI generated from AAL ROI (12).

## Results

 The mean age of the 18 patients (12 females, six males) was 70.6±11.3 years; their mean MMSE score was 22.3±6.8, and their mean ADAS score was 12.5±7.3. Fourteen of the patients had been diagnosed with AD (10 females, four males; mean age: 70.2±12.8 yrs; mean MMSE score: 20.9±6.9; mean ADAS score: 13.6±7.7), and the other four patients had been diagnosed with MCI due to AD (two females, two males; mean age: 73.0±2.2 yrs; mean MMSE score: 27.3±3.1; mean ADAS score: 8.8±4.0). The correlation coefficient between MMSE score and ADAS score was 0.891 (p < 0.001).

**Figure 1:**
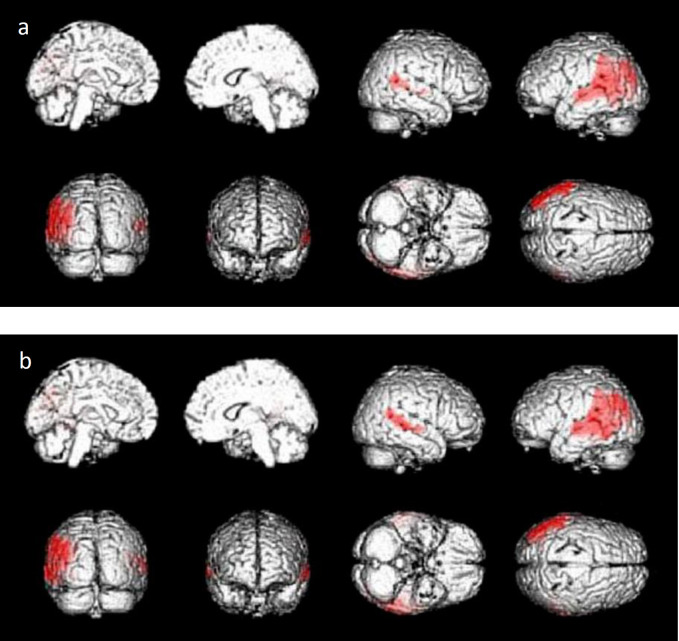
**a**: Areas of significant correlation between the FDG SUVR and the MMSE scores of the 18 patients with AD or MCI due to AD (p<0.001, uncorrected). The bilateral parietotemporal cortices partly including visual cortices are highlighted as significantly correlated areas. **b**: Areas of significant correlation between the FDG SUVR and the patients' ADAS scores (p<0.001, uncorrected). The bilateral parietotemporal cortices partly including visual cortices are highlighted as significantly correlated areas

**Figure 2:**
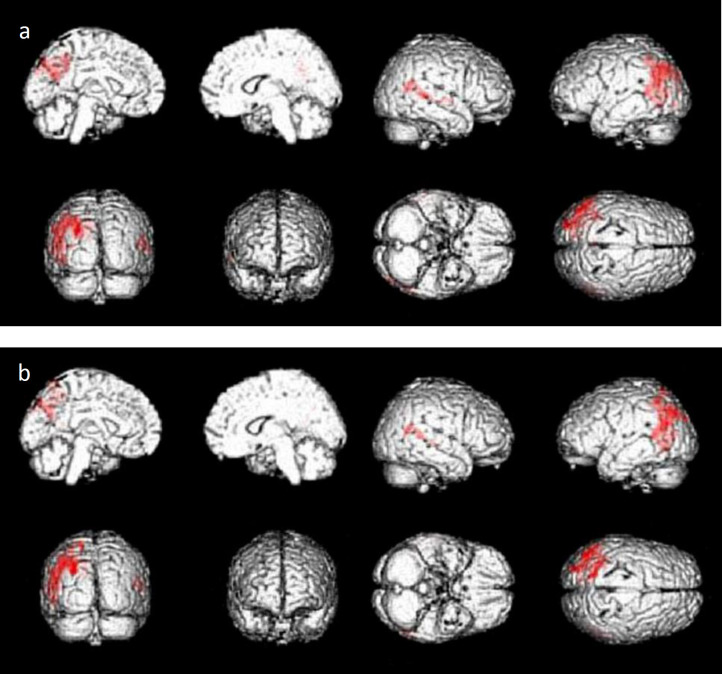
**a**: Areas of significant correlation between the THK5351 SUVR and MMSE scores of the 18 patients with AD or MCI due to AD (p<0.001, uncorrected). The bilateral parietotemporal cortices, partly including visual cortices and the left posterior cingulate/precuneus are highlighted as significantly correlated areas. **b**: Areas of significant correlation between the THK5351 SUVR and the patients'' ADAS scores (p<0.001, uncorrected). The bilateral parietotemporal cortices, partly including visual cortices and the left posterior cingulate/precuneus, are highlighted as significantly correlated areas

 The results of the voxel-wise statistical image analysis are depicted in Figures 1 and 2 and quantified Tables 1 and 2. The FDG SUVR in the bilateral parietotemporal cortices, partly including visual cortices, were significantly positively correlated with the patients' MMSE scores (Figure 1A, Table 1) and significantly negatively correlated with their ADAS scores (Figure 1B, Table 1). 

 There was a negative correlation between the THK5351 SUVR and the patients' MMSE scores and there was a positive correlation between the THK5351 SUVR and their ADAS scores in the bilateral parietotemporal cortices, partly including visual cortices, and the left posterior cingulate gyrus/precuneus (Figure 2, Table 2).

 There were no significant correlations between the regional GM volumes and the MMSE scores or between the regional GM volumes and ADAS scores. There were no significant correlations between the regional PiB SUVR and the MMSE scores or between the regional PiB SUVR and ADAS scores.

**Table 1:** Brain regions in which the FDG SUVR correlated with MMSE and ADAS scores in 18 patients on the AD continuum

**Correlation**	**Brain region**	**Brodmann area**	**MNI coordinates**			**t-value**
			**x**	**y**	**z**	
**FDG-MMSE**	Lt. supramarginal gyrus	40	−62	−40	28	7.62
	Lt. visual association cortex	19	−38	−82	20	6.75
	Rt. angular gyrus	39	50	−46	16	5.05
	Rt. middle temporal gyrus	21	54	−42	6	4.93
**FDG-ADAS**	Rt. angular gyrus	39	48	−48	18	7.07
	Rt. supratemporal gyrus	22	56	−36	6	5.16
	Lt. visual association cortex	19	−36	−78	26	6.54
	Lt. angular gyrus	39	−54	−58	40	5.95

ADAS: Alzheimer Disease Assessment Scale-Cognitive Subtest, FDG: fluorodeoxyglucose, Lt: left, MMSE: Mini-Mental State Examination, MNI: Montreal Neurological Institute, Rt: right, SUVR: standard uptake value ratio

**Table 2:** Brain regions in which the THK5351 SUVR correlates with MMSE and ADAS scores in 18 patients on the AD continuum

**Correlation**	**Brain region**	**Brodmann area**	**MNI coordinates**			**t-value**
			**x**	**y**	**z**	
**THK-MMSE**	Lt. secondary visual cortex	18	−14	−66	24	6.85
	Lt. angular gyrus	39	−48	−60	22	6.58
	Lt. visual association cortex	19	−24	−84	30	6.16
	Rt. middle temporal gyrus	21	64	−38	4	5.35
	Rt. primary auditory cortex	41	56	−10	2	5.22
**THK-ADAS**	Lt. angular gyrus	39	−48	−60	22	5.98
	Lt. posterior cingulate gyrus	31	−16	−64	26	5.11
	Rt. angular gyrus	39	52	−58	16	4.72
	Rt. supratemporal gyrus	22	62	−30	4	4.1

ADAS: Alzheimer Disease Assessment Scale-Cognitive Subtest, FDG: fluorodeoxyglucose, Lt: left, MMSE: Mini-Mental State Examination, MNI: Montreal Neurological Institute, Rt: right, SUVR: standard uptake value ratio, THK: THK5351

Table 3 shows the correlation coefficients between regional FDG SUVR and THK5351 SUVR. There were moderate negative correlations between regional FDG SUVR and THK5351 SUVR: only frontal correlation coefficients was under -0.4 but other parietal, occipital, posterior cingulate/precuneus and temporal correlation coefficients were -0.425 to -0.477.

**Table 3:** Correlation coefficients between regional FDG SUVR and THK5351 SUVR

	**Frontal cortices**	**Occipital cortices**	**Parietal cortices**	**Posterior cingulate/** **Precuneus cortices**	**Temporal cortices**
Correlation coefficient	-0.351	-0.472	-0.425	-0.449	-0.477

## Discussion

 We analyzed the findings of patients who underwent 3D-MRI, FDG-PET, PiB-PET, and THK5351-PET examinations, and we evaluated the correlation between each imaging modality's biomarker results and the patients' scores on cognitive function tests. Concerning regional brain atrophy, there was no significant correlation between the regional GM volumes and MMSE scores or between the regional GM volume and ADAS scores, although another investigation had reported a correlation between medial temporal atrophy and cognitive function test results (13). This discrepancy may have occurred because the number of patients in the present study was not very large (n=18) and the patients' dementia status was relatively mild, as indicated by the mean MMSE score of 22.3±6.8.

 Concerning regional amyloid deposition, we observed no significant correlation between regional PiB accumulations and MMSE scores or between regional PiB accumulations and ADAS scores in this study. This is reasonable because at the MCI stage, amyloid deposition has already reached a near plateau (14). For amyloid PET, PiB was used in this study, but if florbetapir is used as an amyloid PET tracer, a correlation might be detected between occipital florbetapir accumulations and MMSE scores (15).

 In our patient series, bilateral parietotemporal FDG accumulations were correlated with MMSE scores and ADAS scores. This finding is similar to those of previous reports except for the posterior cingulate FDG accumulations described in a recent investigation (16). 

 Regional glucose metabolism demonstrated by FDG-PET reflects cognitive dysfunction, and this is why FDG accumulation is considered a neuronal injury. Our present analyses did not identify posterior cingulate/precuneus FDG uptake, whereas Nobili et al. reported that there were positive correlation between the Memory Complaint Questionnaire (MAC-Q) score and FDG uptake in the bilateral posterior cingulate cortices and left inferior parietal lobule, middle cingulate cortex, precuneus, and angular gyrus (17). This difference in findings may be due to differences in the subjects' characteristics and/or the contents of the neuropsychiatric tests between the MMSE and MAC-Q.

 We observed that regional THK5351 accumulations were correlated with MMSE and ADAS scores in bilateral parietotemporal cortices and the left posterior cingulate gyrus/precuneus. The regions in which the THK5351 accumulations correlate with MMSE and ADAS scores in patients with AD pathology are almost the same as those in which FDG accumulations correlate with MMSE and ADAS scores; an exception is the left posterior cingulate area. These results indicate that although FDG could not show posterior cingulate area as significant area but the tracer THK5351 could on the AD continuum brain.

 Qiao et al. reported that FDG uptake more closely correlated with neuropsychological function compared to ^18^F-AV1451tau PET results (16). We speculate that this is due to differences in the affinity of THK5315 and AV1451 for other substances in addition to tau deposition. Although THK5351 was developed as a tau PET tracer, it was later found to have an affinity not only for tau but also for MAO-B (18), which is one of the markers for imaging astrocytes; its activity is expressed in plaque-associated astrocytes (19). The affinity of THK5351 for MAO-B may limit the utility of THK5351 as a biomarker of tau deposition, but it is possible that THK5351 could be used as a combination tracer of both tau deposition and neuroinflammatory processes. Parietotemporal association cortices and the posterior cingulate gyrus/precuneus are the regions where glucose metabolism is decreased (20) and where tau depositions are seen in brains on the AD continuum (21).

 Sintini et al. reported that a strong negative correlation between AV1451 and FDG-uptake in the frontal and occipital lobes (22), while our study showed there is a moderate negative correlation between FDG SUVR and THK5351 in the parietal, occipital, posterior cingulate/ precuneus and temporal cortices, but not in the frontal cortices. This difference may be due to the difference between the features of sample groups and features of AV1451 and THK5351. If a strong negative correlation can be obtained between FDG and THK5351 across the entire cerebral cortex, it would seem that either PET would be unnecessary. However our study obtained not strong but moderate negative correlations between them, therefore we think both PET studies are necessary for evaluation of AD prognostic status, reflecting different substances.

 A limitation of this study is that severe AD patients were not enrolled because severe AD patients could not undergo all four imaging studies (3D-MRI, FDG-PET, PiB-PET, and THK5351-PET). However, we can say that even within the narrow range of MCI due to AD to mild AD, we observed a strong correlation between regional THK5351 accumulations and neuropsychological test scores.

## Conclusion

 THK5351 correlated as well as or more additional than FDG with neuro-psychological test scores, due to its affinity for both tau deposition and MAO-B in the AD brain. THK5351-PET may thus be useful for monitoring the pathophysiological progression of AD.
